# Efficacy of live B1 or Ulster 2C Newcastle disease vaccines simultaneously vaccinated with inactivated oil adjuvant vaccine for protection of Newcastle disease virus in broiler chickens

**DOI:** 10.1186/1751-0147-48-2

**Published:** 2006-05-26

**Authors:** N Chansiripornchai, J Sasipreeyajan

**Affiliations:** 1 Department of Veterinary Medicine, Faculty of Veterinary Science, Chulalongkorn University, Henri Dunant road, Bangkok 10330, Thailand

## Abstract

Two hundred, one-day-old broiler chicks were divided into groups 1, 2 and 3 containing 60, 70 and 70 chicks, respectively. The groups were divided into subgroups of 10 chicks that were vaccinated according to the following scheme: group 1 unvaccinated control, group 2 vaccinated subcutaneously at 1 day old with inactivated oil adjuvant vaccine (IOAV) in combination with live B1 vaccine. Group 3 was vaccinated in the same mode as group 2 with IOAV and live Ulster 2C vaccine. All birds were challenged when they were 28 days old. Mortality rate, body weight gain and feed conversion ratio (FCR) were monitored before and after challenge. All the chickens in group 1 died, indicating that there was no disease resistance of this unvaccinated control group of chickens. Conversely, the monitored disease resistance of chickens in groups 2 and 3 was 68.57% ± 18.64 and 88.57% ± 9.00, respectively (*P *< 0.05). The morbidity of chickens in groups 2 and 3 was 37.89% ± 14.36 and 14.76% ± 12.40, respectively (*P *< 0.05). The body weight gain, feed intake and FCR of group 3 were significantly better than those of group 2 (*P *< 0.05) during 1–42 days old. The simultaneous vaccination with B1 or Ulster 2C and IOAV of 1-day-old chicks gave some protection of 28-day-old broilers without a booster vaccination.

## Introduction

Newcastle disease (ND), caused by ND virus (NDV) which is an Avulavirus [1] is one of the most important disease encountered in the poultry industry. The first reported ND outbreak occured in 1926 in Java (Indonesia) [2]. ND infection takes place through virus inhalation or ingestion and its spread from one bird to another depends on the availability of the virus in its virulent infectious form [3] and its short incubation period of 5–6 days. The disease normally affects the respiratory, gastrointestinal and nervous systems. Clinical signs associated with ND often begin with listlessness, increased respiration rate and weakness followed later by prostation and death. Morbidity and mortality rates of infected birds vary from 1–100% [4] with the former reaching upto 100% and with the later escalating to 50% in adult birds and 90% in young chickens.

Today there are commercial live and inactivated oil adjuvant vaccines (IOAV) which are very effective as immunization antigens [5]. The live ones are produced from lentogenic and mesogenic virus strains [6]. Live lentogenic strains namely F, Hitchner B1 and La Sota and mesogenic virus strains namely Mukteswar and Komarov are commercially available. The ND Ulster virus strain has been reported to induce a very mild or inapparent disease in susceptible chickens [7-9] after vaccination. Its efficacy of a drinking water-administered and its potential as an aerosol vaccines have been reported by Beard [10] and Gough and Allan [11]. The Ulster strain is known to trigger immunity in the vaccinated chickens with less efficacy than strain Hitchner B1 when given as aerosol vaccine. However, Ulster strain vaccine causes less side effects than B1 [12-15].

Various kinds of live vaccination techniques namely, oral administration through drinking water, course spray, eye-drop, intranasal instillation, subcutaneous and muscle injection have been described [16]. Undesirable vaccination reactions following administration of live ND vaccines are economically important in the broiler industry especially if they result in adverse disease effects (e.g. colibacillosis), growth retardation and sometimes even mortality. On the other hand IOAV based vaccine does not provoke undesirable reactions. However, both types of vaccines and their administration by injection are relatively expensive. In addition, IOAV vaccinated one-day-old broiler chicks which also possess natural maternal antibodies show pronounced immunity between 3 and 4 weeks of age [17].

A combination of live and inactivated vaccination programme is recommended for endemic areas because vaccine combination is known to promote far better immunological protection than administration of only single live vaccine. There are several kinds of vaccination programmes practised in Thailand for broiler chickens. Some of these cause undesirable immunization reactions, which disrupt the level of protective immunity of chicks. As an attempt to circumvent this problem we describe herein a comparative study on vaccination of broiler chicks which were immunized with inactivated vaccine in combination with live B1 or Ulster 2C vaccine strains. B1 and Ulster 2C are vaccinal isolates of NDV, which replicate in the Harderian gland and induce IgA in the tears [18]. Ulster 2C is known to stimulate immunity in the intestinal loop rather than in the respiratory tract and does not give abnormal side effects to the vaccinated chicks [19].

## Materials and methods

### Chickens

Unvaccinated 1-day-old ROSS-308 broiler chicks, obtained from a commercial hatchery were used for the pathogenicity and serology studies. The chicks were maintained in isolation units in the temperature-controlled rooms. They were fed ad lib on commercial poultry feed (Betagro, Bangkok, Thailand). Chickens were identified individually by numbered leg tags. Guidelines and legislative regulations on the use of animals for scientific purposes of Chulalongkorn University, Bangkok, Thailand were followed.

### Strains of Newcastle disease virus

The Ulster 2C (Poulvac-NDW, Fort Dodge Saúde Animal Ltda, Campinas SP, Brazil) and B1 virus strains (Fort Dodge Animal Health, Fort Dodge, Iowa, U.S.A.) used in the experiment were reconstituted from freeze-dried vials. According to the manufacturers' recommendations, each vaccine is in 1000 dose vials, one dose being at least 10^5.7 ^and 10^6.5 ^50% embryo infective dose (EID_50_)/bird, respectively. IOAV (Kimber strain, Chick N-K, Fort Dodge Animal Health, Fort Dodge, Iowa, U.S.A.) was administered subcutaneously at the nape of each chick in 0.1 ml (10^9 ^TCID_50_/bird).

### Challenge study

At 4 wk after vaccination (28 days old) all of the birds were moved to the isolation unit, and each bird was challenged with the viscerotropic velogenic NDV (vvNDV) strain (ICPI = 1.8), at a dose of 10^6 ^EID_50_/bird. Protection was determined for a period of 14 days after challenge. The parameters used to evaluate protection from NDV were birds showing clinical signs and dead birds. This was recorded as morbidity and mortality, respectively.

### Immunization and virus challenge

The live vaccines were administered by coarse spray in a Select laboratory cabinet. The following immunization scheme was applied. Group 1: unvaccinated control group (n = 60 chickens), the broilers received no vaccine. Group 2 (n = 70), the broilers were vaccinated at 1-day-old chicks with strain B1 live vaccine given by course spray in combination with IOAV vaccine administered subcutaneously at the nape of each. Group 3 (n = 70), the broilers were vaccinated at 1-day-old with Ulster 2C by couse spray in combination with IOAV vaccine also administered as in group 2. Chickens in all the 3 groups were further kept in groups of 10 chickens. At 28 days old, all chickens were weighed, amount of feed intake measured for calculation of feed conversion ratio (FCR) and they were all challenged with NDV. Following vaccination and challenge, all chickens were observed for any adverse clinical symptoms (morbidity) and mortality for 2 weeks. At 42 days old, all chickens were weighed and the amount of feed intake was measured from which FCR was determined.

### Statistical analysis

FCR were analyzed and compared between groups using an independent Student's t-test with SPSS 9.0 software. Morbility and mortality were calculated by using Chi-square values. Differences between groups were considered significant at *p *< 0.05.

## Results

### The protective efficacy of different NDV strains

All chickens in group 1 died during 28–42 days old after challenge. The clinical signs in affected birds included gasping, paralysis and torticollis. The autopsy lesions confirmed Newcastle disease virus infection depicted by hemorrhage in proventriculus, small intestine, caecal tonsil, rectum, heart muscle, coronary fat and pneumonitis. The mortality rate of chickens in the vaccinated groups 2 (31.43 ± 18.64) and 3 (11.43 ± 9.00) were significantly different (*P *< 0.05) and it was lower than those for unvaccinated control chickens (100.00 ± 0.00) (*P *< 0.05). The dead chickens in groups 2 and 3 were found to have ND lesions but not as many as those found in unvaccinated control group 1. The disease resistant percentage of chickens in groups 1, 2 and 3 was 0.00, 68.57 ± 18.64 and 88.57 ± 9.00, respectively with statistical significance of *P *< 0.05. Percentage of morbidity of live birds of groups 2 and 3 at 2 weeks after challenge was 37.89 ± 14.36 and 14.76 ± 12.40, respectively (*P *< 0.05).

### The effect of NDV vaccine on weight gain and FCR

The results of weight gain, feed intake and FCR are shown in table 1. At 1–28 days old, the weight gain of chickens in the vaccine groups was not significantly different from that of the control group. The feed intake between the vaccine groups was significantly different (*P *< 0.05) but it was not significantly different from that of the control group. The FCR of the chickens in group 2 revealed the best among all groups. At 28–42 days old, the weight gain and feed intake of chickens in the vaccine groups were significantly diffferent (*P *< 0.05). The FCR of group 2 could not be calculated because it was in the negative value. At 1–42 days old, the body weight gain, feed intake and FCR of group 3 were significantly better than those of group 2 (*P *< 0.05).

**Table 1:** Body weight gain, feed intake and FCR of chickens at 1–28, 28–42 and 1–42 days old (Mean ± SD)

Group	chickens age 1–28 days old			chickens age 28–42 days old			chickens age 1–42 days old		
	
	body weight gain (kg)	Feed intake (kg)	FCR	body weight gain (kg)	Feed intake (kg)	FCR	body weight gain (kg)	Feed intake (kg)	FCR
1 (n = 6)	9.62 ± 0.78^a^	15.12 ± 0.41^a,b^	1.55 ± 0.11^a^	-^#^	-	-	-	-	-
2 (n = 7)	10.08 ± 0.57^a^	14.73 ± 0.74^a^	1.46 ± 0.03^b^	0.67 ± 2.73^a^	12.28 ± 1.12^a^	^##^	10.75 ± 1.44^a^	27.02 ± 1.68^a^	2.51 ± 0.09^a^
3 (n = 7)	10.25 ± 0.49^a^	15.54 ± 0.38^b^	1.52 ± 0.07^a,b^	3.20 ± 1.71^b^	14.11 ± 1.06^b^	5.59 ± 2.88	14.68 ± 1.00^b^	29.65 ± 1.26^b^	2.02 ± 0.06^b^

^#^All chickens died.

^##^The values cannot be calculated because of minus value

The different superscript in each colume means statistically significant difference (*p *< 0.05)

## Discussion

It is well known that a combination of live and inactivated oil adjuvant Newcastle disease vaccines could elicit the protection against vvNDV challenge [5]. So, we would like to compare the disease resistance of chickens against vvNDV by using different strains of live virus vaccine vaccinated at 1 day old. In this experiment it was found that all chickens in the non-vaccinated group 1 died after challenge. Chickens in groups 2 (vaccinated with IOAV and B1 strain live vaccine) and 3 (vaccinated with IOAV and Ulster 2C strain live vaccine) exhibited resistance after challenge. The chickens in group 3 revealed the protection from the vvNDV better than the chickens in group 2. According to *Folitse et al *[20], the reason for the high antibody response obtained with killed-in-oil plus live virus vaccines given simultaneously could be that initially, the live virus replicates quickly in the mucosal membrane of the conjunctiva and the nostrils, eliciting a primary immune response. This is followed by a continuous slow release of the killed virus antigen trapped in the oil medium, thus allowing the killed virus antigen to behave as a booster dose. *Russell *[18] and *Kohn and Ebert *[19] who showed that Ulster 2C strain can replicate in the Harderian gland and induce IgA in the tears and also it is known to stimulate immunity in the intestinal loop that can elicit higher HI titers comparing to Hitchner B1 strain. According to our experiment, once vaccination at one day old cannot give sufficient immunity to protect chickens until slaughter at 42 days old. The results reveal that chickens in group 2 had 68.57 ± 18.64% disease resistance because the live B1 strain vaccine evoked local immunity in the respiratory tract [21] which develops 2 days after vaccination. This response comes from the cell-mediated response [16]. *Zakey-Rones and Levy *[22] pointed out that a local immunity response which has previously been shown to be evoked by local administration of antigen might be unaffected by the presence of maternal antibodies. The live Ulster 2C strain virus vaccine, which mainly multiplies in the intestinal loop such as caecal tonsil and rectum but it also, multiplies in the epithelial cells of the respiratory tract [19,23] that triggers higher immune response. It is accordance with the results of disease resistance and morbidity rate of chickens in group 3 are better than chickens in groups 2. These results are in agreement with the work of *van Eck et al*. [14] who showed that chickens immunized with Ulter 2C strain vaccine give a better response than chickens vaccinated with B1 strain when exposed to the vaccines at 1–10 days of age. Another reason for the differences in the response could be related to the secondary (anamnestic or recall) response which indicates potential virulence of the virus strain used in the challege. Chickens in group 3, received Ulster 2C, had lower the morbidity and mortality than chickens in group 2. Under the conditions of the experiment reported here, it has been shown that simultaneous vaccination with B1 and IOAV, and Ulster 2 C and IOAV of 1-day-old chicks with maternally derived antibodies gave some protection of 28-day-old broilers without a booster vaccination. Live birds of group 2 depicted higher morbidity rate than birds of group 3. Thus the carcass quality of chickens vaccinated with B1 strain was lower than that of birds exposed to Ulster 2C strain.

Chickens in group 2 at 1–28 days old (before challenge) had the best FCR. They were followed by chickens in groups 3 and 1. This is in agreement with *van Eck and Goren *[13] who showed that Ulster 2C vaccine retards growth of chickens that have high maternal immunity. At 28–42 days old (after challenge), the body weight gain and feed intake chickens in group 3 had statistically significant difference (*P *< 0.05) of better than chickens in group 2 because the mortality rate of chickens in group 2 was higher than that of chickens in group 3. Anyhow, during 1–42 days old, the body weight gain, feed intake and FCR of group 3 were significantly better than those of chickens in group 2 (*P *< 0.05).

## Abbreviations

ND = Newcastle disease, NDV = Newcastle disease virus, vvNDV = viscerotropic velogenic Newcastle disease virus, FCR = Feed conversion ratio, ICPI = Intracerebral Pathogenicity Index, IOAV = Inactivated Oil Adjuvant Vaccine
